# Advances in research on the effects of medicinal and edible substances on lung cancer: an updated review

**DOI:** 10.3389/fphar.2025.1735406

**Published:** 2026-01-22

**Authors:** Ying Yang, Lingli Jiang, Ke Li, Genming Zhang, Junpeng Wang, Daorui Li

**Affiliations:** 1 Oncology Department, Guang’anmen Hospital of China Academy of Chinese Medical Sciences, Beijing, China; 2 Graduate School, Beijing University of Chinese Medicine, Beijing, China; 3 General Surgery Department, Guang’anmen Hospital of China Academy of Chinese Medical Sciences, Beijing, China

**Keywords:** active ingredients, lung cancer, mechanism, medicinal and edible substances, traditional Chinese medicine

## Abstract

Lung cancer, as one of the malignant tumors with the highest morbidity and mortality worldwide, poses a serious threat to human life and health. Despite the fact that there have been significant advances in the modern medicine treatment of lung cancer, the problem of drug resistance and the potential toxic side effects associated with the respective treatment still restrict the clinical efficacy and the amelioration of patients’quality of life. Based on this background, the therapeutic value of traditional Chinese medicine (TCM) as an adjuvant has become increasingly prominent. Among these, medicinal and edible substances have garnered growing research attention for the adjuvant treatment of lung cancer, owing to their wide sources, fewer side effects, and multi-pathway anti-tumor potential. Medicinal and edible substances abound with polysaccharides, flavonoids, terpenoids, saponins, polyphenols, and other bioactive substances. They are pivotal in the prevention and treatment of lung cancer through multiple mechanisms, including anti-inflammatory effects, antioxidant effects, immunomodulation effects, inhibition of tumor angiogenesis effects, and cell regulation effects. In this review, the pharmacological action of active components in medicinal and edible substances and the mechanism of action in the treatment of lung cancer were reviewed systematically, and the research direction of medicinal and edible substances in the field of lung cancer in the future was prospected, in an effort to provide theoretical reference for promoting its in-depth application in the comprehensive treatment of tumors, as well as for its complementarity of strengths and coordinated advancement with modern medicine.

## 1 Introduction

Lung cancer (LC) is a malignant neoplasm type with high incidence worldwide, characterized by high mortality, insidious onset, and poor prognosis (Rina et al., 2024). Based on updated estimates from *GLOBOCAN*, LC was the most frequently diagnosed cancer in 2022, responsible for almost 2.5 million new cases and an estimated 1.8 million deaths (Bray et al., 2024). LC has ranked first globally in terms of incidence and mortality among all cancers for consecutive years. While cigarette smoking represents the most significant risk factor for LC, other elements such as age, gender, dietary habits, pre-existing lung conditions, environmental pollution, and occupational exposures also play crucial contributing roles (Feng et al., 2025a). For the early-stage LC patients, the standard care is surgical resection. However, even after surgical resection, the 5-year survival rate is only 38%–60% (Dennehy et al., 2025). Current mainstay therapies for patients with surgically unresectable LC include radiotherapy, chemotherapy, targeted therapy, and immunotherapy. Although these modalities are demonstrated to be efficacious in disease control, they are often limited by considerable adverse reactions and toxic side effects, including alopecia, diarrhea, and bone marrow suppression (Liu et al., 2021), thereby severely compromising patients’quality of life and treatment compliance. In recent years, due to the strong anticancer effect and small toxicity of medicinal and edible substances, the application in cancer adjuvant therapy has increased significantly (Xi et al., 2025), and has gradually become an important supplementary strategy in the research and development of anti-tumor drugs and clinical practice.

Medicinal and edible substances, also known as “food as medicine”, represents a fundamental concept and philosophical basis in Traditional Chinese Medicine (TCM). Its approach underscores that certain foods possess a dual function, serving as both essential nutrient sources and therapeutic agents. In practice, medicinal and edible substances can be consumed as part of a normal diet while exerting defined pharmacological properties (Wang et al., 2025). Dietary therapy with these substances can enhance nutrient intake, help alleviate patient symptoms, and improve overall constitution. Although rooted in TCM, the concept of medicinal and edible substances has recently gained global attention, especially through its integration of preventive medicine with nutritional therapy. This perspective has revitalized worldwide research and development in the functional food sector. Substantial modern studies confirm that medicinal and edible substances possess multiple bioactive properties, including anti-inflammatory, antioxidant, blood glucose-lowering, anti-cancer, and immunomodulatory effects. They are already utilized in managing various conditions such as cardiovascular diseases (Jin et al., 2024), kidney diseases (Zhao et al., 2024), diabetes (Gong et al., 2020), and pancreatitis (Yang et al., 2021). Furthermore, accumulating evidence indicates that certain medicinal and edible substances can enhance the efficacy of conventional cancer therapies, reduce adverse effects, improve patient symptoms, and potentially lead to improved survival and clinical outcomes in cancer patients (Wang et al., 2025). Building upon reported progress in gastric cancer research (Hao et al., 2025), this article will preliminarily explore the research advances regarding the impact of medicinal and edible substances on LC.

**FIGURE 1:**
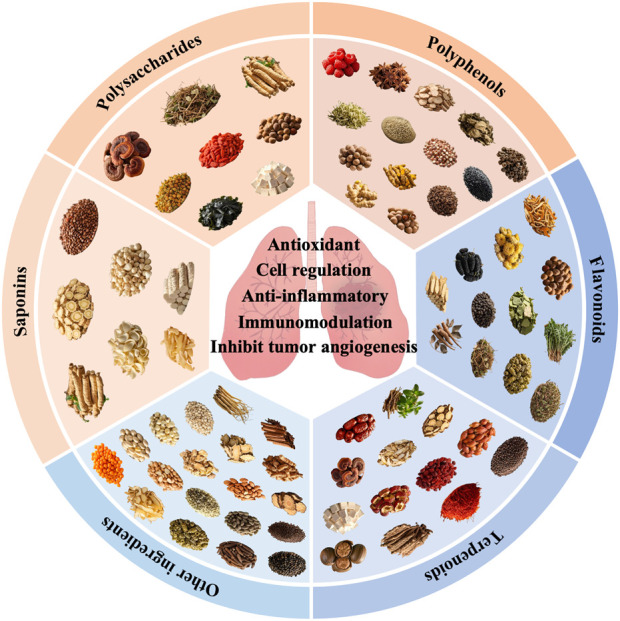
Classification of medicinal and edible substances by active ingredients.

As of now, the official medicinal and edible substances list includes 106 medicinal herbs, each abundant in bioactive compounds that play significant physiological roles. Through retrieval and systematic analysis of extensive literature from authoritative databases such as PubMed and Web of Science, medicinal and edible substances with reported effects on LC have been categorized by active ingredient, as shown in Figure 1. These are broadly classified into polysaccharides, flavonoids, terpenoids, saponins, polyphenols, and other ingredients. Current evidence suggests that these compounds may exert potential effects in LC prevention and treatment through various mechanisms, including anti-inflammatory effects, antioxidant effects, immunomodulation effects, inhibiting tumor angiogenesis effects, and cell regulation effects (summarized in Table 1 by mechanism type). The objective of this review is to systematically summarize the active components, pharmacological activity, and mechanisms by which medicinal and edible substances influence LC.

**TABLE 1:** Mechanisms of medicinal and edible substances in the prevention and treatment of LC.

Main mechanism	TCM extract/monomer	TCM source	Family	Classification	Mediated pathway	References
Cell regulation effecets	Laminaria japonica polysaccharides	*Lonicera japonica Aresch*	Laminariaceae	Polysaccharides	Regulating inflammatory responses, cell apoptosis, and cell cycle progression by directly binding to key proteins such as PPARg, PTGS2, CASP3, BCL2, and CCND.	Jin W. et al. (2025)
​	HCA4S1	*Houttuynia cordata Thunb*	Saururaceae	Polysaccharides	Upregulating the expression levels of cleaved caspase 3 and cyclin B1, thereby inducing cell cycle arrest and apoptosis	Han et al. (2018)
	*Poria cocos* wolf extract	*Poria cocos (Schw.) Wolf*	Polyporaceae	Polysaccharides	Increasing the cleaved forms of caspase-3 and poly (ADP-ribose) polymerase (PARP), thereby inducing apoptosis in human lung cancer cells	Qiu et al. (2024)
	GQZ lipopolysaccharide	*Lycium barbarum L*	Solanaceae	Polysaccharides	Inducing apoptosis and inhibiting the proliferation of NSCLC *in vitro* and *in vivo* by inhibiting the PI3K/AKT1 signaling pathway	Zhang et al. (2022)
	Curcumin	*Curcuma longa L*	Zingiberaceae	Polysaccharides	Targeting cancer cells selectively, enhancing cytotoxicity against lung cancer cells compared to alone	Cho et al. (2024)
	*Lonicera japonica* Polyphenolic compounds	*Lonicera japonica Thunb*	Caprifoliaceae	Polyphenols	Inducing apoptosis by regulating the protein expression levels of caspases, poly-(ADP-ribose) polymerase, and the Bax/Bcl-xL	Park et al. (2017)
	Cannabidiol	*Cannabis sativa L*	Cannabaceae	Polyphenols	Inducing tumor cell apoptosis by the activation of the ERK, PI3K, p38MAPK, and ceramide pathways	Nigro et al. (2021); Ramer et al. (2010)
	Eugenol	*Syzygium aromaticum* (L.) Merr. and L.M.Perry	Myrtaceae	Polyphenols	Targeting β-catenin to inhibit its nuclear transport, induce cytoplasmic degradation via N-terminal Ser37 phosphorylation, downregulate CSC markers, enhance apoptosis and suppress cell proliferation	Choudhury et al. (2021)
	Phyllanthus emblica extract	*Phyllanthus emblica L*	Phyllanthaceae	Polyphenols	Releasing iron ions in lysosomes, generating reactive oxygen species *via* chemical reactions, intensifying DNA damage, and promoting cancer cell apoptosis	Thoidingjam and Tiku (2019)
​	Sesamol	*Sesamum indicum L*	Pedaliaceae	Polyphenols	Increasing the activity of caspase 8, 9, and 3/7 inhibites SK-LU-1 cell growth, indicating that apoptotic cell death occurred through both extrinsic and intrinsic pathways	Siriwarin and Weerapreeyakul (2016)
​	Kaempferol	*Kaempferia galanga L*	Zingiberaceae	Polyphenols	Regulating EGFR/PI3K/AKT and IGF1R/PI3K/AKT signaling pathways by targeting EGFR, IGF1R, PIK3R1 and Akt1	Zhang J. et al. (2023)
​	Myristicin	*Myristica fragrans Houtt*	Myristicaceae	Polyphenols	Mediating apoptotic cell death was confirmed by MMP loss, increasing ROS, cleaving PARP, and decreasing pro-caspase3	Maheswari et al. (2018)
​	E. ferox Salisb extract	*Euryale ferox Salisb*	Nymphaeaceae	Polyphenols	Inducing apoptosis by inhibiting the Akt protein and activating the p53 protein	Nam et al. (2019)
​	Lotus Leaf Flavonoids	*Nelumbo nucifera Gaertn*	Nelumbonaceae	Flavonoids	Upregulating the expression of p38 MAPK, caspase-3、9, cleaved caspase-3、9, and Bax and downregulating the expression of Cu/Zn SOD, CAT, Nrf2, NQO1, HO-1, and Bcl-2 in A549 cells	Jia et al. (2021)
​	Puerarin 6″-O-xyloside	*Pueraria montana var. lobata (Willd.) Maesen and S.M.Almeida ex Sanjappa & Predeep*	Fabaceae	Flavonoids	Inhibiting the Akt/c-Myc signaling pathway activation, impairing cell viability, suppressing self-renewal and invasiveness, and downregulating CD133, CD44, and ALDH1 mRNA expression	Tao et al. (2020)
​	Linarin	*Chrysanthemum × morifolium (Ramat.) Hemsl.*	Asteraceae	Flavonoids	Downregulating MMP-9 and suppressing NF-κB activation by inhibiting NF-κB and IκB-α phosphorylation	Jung et al. (2019)
​	Quercetin and kaempferol	*Portulaca oleracea L*	Portulacaceae	Flavonoids	Downregulating Bcl-2, upregulating Bax, activating caspase-3/9 to induce cancer cell apoptosis, inhibiting PI3K/Akt and ERK1/2 pathways, and reducing VEGF signaling	Khazdair et al. (2021)
​	Quercetin	*Dendrobium officinale Kimura & Migo*	Orchidaceae	Flavonoids	Inducing pro-apoptotic autophagy *via* the SIRT1/AMPK signaling pathway	Guo et al. (2021)
​	CAVAPs	*Citrus grandis ‘Tomentosa'*	Rutaceae	Flavonoids	Activating macrophages through the MAPK and NF-κB signaling pathway, stimulating the production of TNF-α and IL-6, and scavenging DPPH radicals	Shen et al. (2017b)
​	β-sitosterol	*Raphanus sativus L.*	Brassicaceae	Terpenoids	Inhibiting miR-181a-3p to upregulate the expression of its downstream target SHQ1, activates the SHQ1/UPR signaling pathway	Wang M. et al. (2024)
​	Glycyrrhetinic acid	*Glycyrrhiza uralensis Fisch*	Fabaceae	Terpenoids	Targeting Caspase-3 and Peroxiredoxin 6, inducing intracellular ROS accumulation, activating the mitochondrial apoptosis pathway to inhibit tumor cell proliferation and promote apoptosis	Guo et al. (2024)
​	Crocin	*Crocus sativus L*	Iridaceae	Terpenoids	Increasing the mRNA levels of both p53 and Bax, decreasing Bcl-2 mRNA expressions, and inducing apoptosis in A549 and SPC-A1 cells, accompanied by an increase in G0/G1 arrest	Chen et al. (2015)
​	Maslinic acid	*Crataegus pinnatifida Bge*	Rosaceae	Terpenoids	Inhibiting proliferation and inducing apoptosis by suppressing caspase-3/8/9, and increasing cleaved caspase-3/8/9, increasing Smac, and decreasing c-IAP1, c-IAP2, XIAP, and Survivin	Bai et al. (2016); Yu et al. (2021)
​	Cornus officinalis extract	*Cornus officinalis Siebold & Zucc*	Cornaceae	Terpenoids	By the MTT method, the cornus officinalis extract showed weak inhibitory activity against the lung cancer A549 cell line	Li et al. (2015)
​	Mogrol	*Siraitia grosvenorii (Swingle) C.Jeffrey ex A.M.Lu & Zhi Y.Zhang*	Cucurbitaceae	Terpenoids	Inducing the excessive autophagy and autophagic cell death *via* activating the AMPK signaling pathway, as well as cell cycle arrest and apoptosis *via* activating the p53 pathway	Li et al. (2022)
​	Lactucin	*Cichorium intybus L*	Asteraceae	Terpenoids	Downregulating the MAPK pathway, cyclin, and cyclin-dependent kinases, inhibiting DNA repair while upregulating p53, p21, Bax, PTEN, and downregulating Bcl-2	Imam et al. (2022)
​	Ursonic acid	*Ziziphus jujuba Mill*	Rhamnaceae	Terpenoids	Inhibiting the expression of MMP-2, -9 by suppressing the ERK and CREB signaling pathways, and reducing the mRNA levels of MMP-1by inhibiting the ERK and c-Fos signaling pathways	Son and Lee (2020)
​	Lily saponin	*Lilium lancifolium Thunb*	Liliaceae	Saponins	Inhibiting intracellular DNA synthesis through reducing the expression of PCNA, and inducing apoptosis by regulating the expression of Bcl-2 and Bax proteins	Luo et al. (2018); Zhou et al. (2024)
​	Furostanol Saponins	*Asparagu cochinchinensis (Lour.) Merr*	Asparagaceae	Saponins	Inhibiting cell proliferation and inducing cytotoxicity, thereby suppressing the growth of lung cancer cells	Zhang R. S. et al. (2021)
​	Furostanol saponins: Macrostemonoside E、F	*Allium chinense G.Don*	Amaryllidaceae	Saponins	Downregulating the anti-apoptotic protein Bcl-2, upregulating the pro-apoptotic protein Bax, and activating the caspase cascade, they induce G2/M cell-cycle arrest and initiate apoptosis	Wang Y. et al. (2019)
​	Ophiopogonin B	*Ophiopogon japonicus (Thunb.) Ker Gawl*	Asparagaceae	Saponins	Reducing migration and invasion by strengthening the Axin/β-catenin interaction and reducing β-catenin protein translocation, downstreaming cyclin D1 and c-Myc	Zhang S. et al. (2021)
​	Ginsenoside Rb1	*Panax quinquefolius L*	Araliaceae	Saponins	Inducing apoptosis by altering the levels of P53, Bax, Cyto-c, Caspase-8, Caspase-3, Cleaved Caspase-3, Bcl-2, MMP-2, and MMP-9 proteins and activating the external apoptotic pathway	Feng L. et al. (2024)
​	Jujuboside B	*Ziziphus jujuba Mill*	Rhamnaceae	Saponins	Increasing the expression of Nox4 and ATF3, elevating the levels of MDA and ROS, as well as reducing the expression of SLC7A11 and Gpx4, and the level of GSH.	Kim and Ko (2025)
​	Seabuckthorn Pulp Oil	*Hippophae rhamnoides L*	Elaeagnaceae	Other ingredients	Triggering autophagic cell death and senescence against cancer cells as a result of sustained ERK phosphorylation and intracellular ROS production in NSCLC.	Batbold and Liu (2022)
​	Polygonatum cyrtonema lectin	*Polygonatum cyrtonema Hua*	Asparagaceae	Other ingredients	Generating ROS and ROS scavenger NAC, activating MAPK members ERK, JNK, and p38, JNK inhibitor, and p38 inhibitor partially reduce PCL-induced apoptosis and autophagy	Liu et al. (2016)
​	Neferine	*Nelumbo nucifera Gaertn*	Nelumbonaceae	Other ingredients	Elevating ROS and reducing BCL2/BAX ratio, targeting ROCK 1, inhibiting the invasion, metastasis, EMT process, and attenuating EMT-related changes of E-cadherin and N-cadherin	Hu et al. (2023)
​	Cinnamon Twig Essential Oil	*Cinnamomum verum J.Presl*	Lauraceae	Other ingredients	Arresting the cell cycle, increasing ROS accumulation, causing mitochondrial depolarisation, and elevating caspase-3/8/9, involved in apoptosis, TNF, IL17, and MAPK signalling pathways	Mohanty et al. (2024)
​	Sword Bean Extract	*Canavalia gladiata (Jacq.) DC.*	Fabaceae	Other ingredients	Inhibiting A549 cell proliferation dose-dependently, reducing ascites, solid tumor growth, intracellular GSH, and restoring abnormal hematological parameters	Abeesh et al. (2021)
​	Echinacoside	*Cistanche deserticola Ma*	Orobanchaceae	Other ingredients	Inducing Mitochondria-Mediated Pyroptosis through Raf/MEK/ERK Signaling	Tang Y. et al. (2024)
​	Coixol	*Coix lacryma-jobi var. ma-yuen (Rom.Caill.) Stapf*	Poaceae	Other ingredients	Blocking the G_2_ phase of the cell cycle, activating p38 MAP kinase, releasing mitochondrial cytochrome c, and activating caspases to induce apoptosis	Lee et al. (2008); Wang X. C. et al. (2024)
​	Bibenzyl extract	*Dendrobium officinale Kimura & Migo*	Orchidaceae	Other ingredients	Inhibiting cancer cell proliferation and modulating the PI3K-Akt signaling pathway	Guo et al. (2021)
​	Black Pepper Extracts	*Piper nigrum L*	Piperaceae	Other ingredients	Targeting cancer cells selectively, enhancing cytotoxicity against lung cancer cells compared to the use alone	Cho et al. (2024)
​	Lobetyolin and lobetyol	*Codonopsis lanceolata (Siebold & Zucc.) Benth. and Hook.f. ex Trautv*	Campanulaceae	Other ingredients	Reducing Ras, PI3K, p-AKT, Bcl-2, cyclin D1, and CDK4 but increasing the expression of Bax, GSK-3β, and clv-caspase-3/9, which could be reversed by the PI3K activator 740 YP.	Wang M. C. et al. (2022)
​	Alpinia officinarum extract	*Alpinia officinarum Hance*	Zingiberaceae	Other ingredients	Inhibiting the proliferation by exhibiting a dose-dependent cytotoxic effect	Alasmary et al. (2019)
​	The extract of Foeniculum vulgare Mill	*Foeniculum vulgare Mill*	Apiaceae	Other ingredients	Inhibiting Bcl-2 protein expression, reducing mitochondrial membrane potential, and releasing Cytochrome C., inhibiting colony formation and cell migration	Ke et al. (2021)
Anti-inflammatory effects	Anethole and shikimic acid	*Illicium verum Hook.f*	Schisandraceae	Polyphenols	Decreasing levels of MDA, p53, TNF-α, and fibronectin, and decreasing cell viability	Abdelaziz et al. (2024)
​	Menthol	*Mentha haplocalyx Briq*	Lamiaceae	Terpenoids	Modulating MAPK and PI3k/Akt pathways, upregulating Bax and p53 genes, and modulating of TNF, IL-6, IFN-γ, and IL-8	Tafrihi et al. (2021)
​	Catalpol	*Rehmannia glutinosa (Gaertn.) Libosch. ex DC.*	Orobanchaceae	Terpenoids	Inhibiting the TGF-β1-induced cell migration and invasion, MMP2, MMP9 repressing the EMT process, activating Smad2/3 and NF-κB signaling pathways	Wang Z. et al. (2019)
​	Dioscin	*Dioscorea oppositifolia L*	Dioscoreaceae	Saponins	Reducing in the expression of p-AKT, MMP2, and PCNA, reducing the expression of p-AKT, MMP2, PCNA and increasing the expression of active-caspase3	Xi et al. (2022)
​	Amygdalin	*Prunus armeniaca L*	Rosaceae	Other ingredients	Enhancing the expression of NF-κB-1 and inactivating NF-κB signaling cascade, and further changing the expressions of proteins Bax, Bcl-2, cytochrome C, caspase 3/9, and PARP.	Lin et al. (2022)
​	Polygonatum odoratum lectin	*Polygonatum odoratum (Mill.) Druce*	Asparagaceae	Other ingredients	Inhibiting the Akt-NF-κB pathway triggered autophagy *via* suppressing the Akt-mTOR pathway	Li et al. (2014)
​	Perilla Seed Oil	*Perilla frutescens (L.) Britton*	Lamiaceae	Other ingredients	Decreasing the levels of IL-1β, IL-6, IL-8, TNF-α, and COX-2, scavenging TNF-α induced ROS levels, decreasing the MnSOD, FOXO1, NF-κB, and JNK signaling pathway	Tantipaiboonwong et al. (2021)
​	Piperlongumine	*Piper longum L*	Piperaceae	Other ingredients	Inducing endoplasmic reticulum stress, which inhibits macrophage M2-type polarization and reduces cell migration	Zhou et al. (2025)
Antioxidant effects	Star Anise Extract	*Star Anise (Illicium verum)*	Schisandraceae	Polyphenols	Decreasing levels of MDA, p53, TNF-α, and fibronectin, and decreasing cell viability	Abdelaziz et al. (2024)
​	Raspberry seed extract	*Rubus chingii Hu*	Rosaceae	Polyphenols	Scavenging hydroxyl and superoxide radicals, which are key players in cancer development, selectively inhibiting the growth of lung cancer A-549 cells	Simonovic et al. (2021)
​	Longan Pericarp-derived Phenolics	*Dimocarpus longan Lour*	Sapindaceae	Polyphenols	Exhibiting DPPH radical scavenging capacity, hydroxyl radical (•OH) inhibitory activity, and exerting the strongest inhibitory effect on the growth of A549 cells	Bai et al. (2019)
​	Chlorogenic acid	*Morus alba L*	Moraceae	Polyphenols	Scavenging activity against DPPH radicals, selectively inhibiting the growth of MCF-7 cells	Srisomsap et al. (2024)
​	Hesperidin	*Citrus reticulata Blanco*	Rutaceae	Flavonoids	Downregulating the expression of MMPs, inhibiting tumor cell proliferation, and inhibiting proliferation and promotion of apoptosis through the miR-132/ZEB2 signaling pathway	Balakrishnan and Menon (2007); Kamaraj et al. (2009); Tan et al. (2020)
​	Ononin	*Astragalus mongholicus Bunge*	Fabaceae	Flavonoids	Inhibiting the growth of lung cancer cells, inducing apoptosis, and suppressing the excessive activation of the HIF-1α/VEGF pathway	Zhang Y. M. et al. (2024)
​	Quercetin and kaempferol	*Portulaca oleracea L*	Portulacaceae	Flavonoids	Downregulating Bcl-2, upregulating Bax, activating caspase-3/9 to induce cancer cell apoptosis, inhibiting PI3K/Akt and ERK1/2 pathways, and reducing VEGF signaling	Khazdair et al. (2021)
​	Perilla Seed Oil	*Perilla frutescens (L.) Britton*	Lamiaceae	Other ingredients	Decreasing the levels of IL-1β, IL-6, IL-8, TNF-α, and COX-2, scavenging TNF-α induced ROS levels, decreasing the MnSOD, FOXO1, NF-κB, and JNK signaling pathway	Tantipaiboonwong et al. (2021)
​	Amygdalin	*Persicae Semen (Taorenh)*	Rosaceae	Other ingredients	Targeted oncogenic and tumor-suppressive pathways, including PI3K-Akt, MAPK, TNF, Ras, focal adhesion, and HIF-1	Lee et al. (2021)
Immunomodulation effects	CAVAPs	*Citrus maxima (Burm.) Merr*	Rutaceae	Flavonoids	Activating macrophages through the MAPK and NF-κB signaling pathway, stimulating the production of TNF-α and IL-6, and scavenging DPPH radicals	Shen et al. (2017b)
​	Ginsenoside Rb1	*Panax quinquefolius L*	Araliaceae	Flavonoids	Inducing apoptosis by altering the levels of P53, Bax, Cyto-c, Caspase-8, Caspase-3, Cleaved Caspase-3, Bcl-2, MMP-2, and MMP-9 proteins and activating the external apoptotic pathway	Feng M. et al. (2024)
​	CAVAPs	*Citrus × aurantium L*	Rutaceae	Flavonoids	Activating RAW264.7 macrophages *via* MAPK and NF-κB signaling pathways, stimulating TNF-α and IL-6, and promoting iNOS, TNF-α, IL-1β, and IL-6, regulating the immune system	Shen et al. (2017a)
​	Total Flavonoids from Taraxacum Mongolicum	*Taraxacum mongolicum Hand.-Mazz*	Asteraceae	Flavonoids	Increasing CD4^+^, CD8^+^, and CD4^+^/CD8^+^, increasing IL-2, IL-3, IFN-γ, and TNF-α, and reducing Ki67, and improving the host’s protective immune response	Kang et al. (2021)
​	6-gingerol	*Zingiber officinale Roscoe*	Zingiberaceae	Polyphenols	Inhibiting the expression of the pathway activation, as well as the expression of PD-L1, and the regulation of iron metabolism, and the modulation of p53 expression	Kang et al. (2023); Yu et al. (2020)
​	Moracin N	*Morus alba L. leaves*	Moraceae	Polyphenols	Targeting the PD-L1/PD-1 signaling pathway, downregulate its expression and block PD-L1/PD-1 binding, thereby enhancing T cell-mediated immunity, development and metastasis of lung cancer	Ye et al. (2024)
​	Curcumin	*Curcuma longa L*	Zingiberaceae	Polysaccharides	Inhibiting JAK2/STAT3, and activating tumor-suppressor genes (RAR-β),downregulating oncogenic miR-21, miR-186, upregulating tumor-suppressive miR-192-5p, miR-215, miR-874	Cho et al. (2024)
​	Pectin HCA4S1	*Houttuynia cordata Thunb*	Saururaceae	Polysaccharides	Upregulating the expression levels of cleaved caspase 3 and cyclin B1, thereby inducing cell cycle arrest and apoptosis	Han et al. (2018)
​	Platycodin D	*Platycodon grandiflorus (Jacq.) ADC.*	Campanulaceae	Saponins	Increasing caspase-3 and PARP immunopositive cells, enhancing iNOS and TNF-α immunoreactivity, and reducing COX-2 immunoreactivity in tumor tissues	Park et al. (2014); Feng L. et al. (2024)
​	Piperlongumine	*Piper longum L*	Piperaceae	Other ingredients	Inducing endoplasmic reticulum stress, which inhibits macrophage M2-type polarization and reduces cell migration	Zhou et al. (2025)
Inhibit tumor angiogenesis effects	Ganoderma lucidum polysaccharides	*Ganoderma lucidum (Curtis) P. Karst*	Ganodermataceae	Polysaccharides	Downregulating EGFR expression, inhibiting PI3K/AKT/mTOR, ERK, Wnt/β-catenin, and suppressing EMT and angiogenesis	Zhang et al. (2025)
​	Ononin	*Astragalus mongholicus Bunge*	Fabaceae	Flavonoids	Inhibiting the growth of lung cancer cells, inducing apoptosis, and suppressing the excessive activation of the HIF-1α/VEGF pathway	Zhang Y. M. et al. (2024)
​	Quercetin and kaempferol	*Portulaca oleracea L*	Portulacaceae	Flavonoids	Downregulating Bcl-2, upregulating Bax, activating caspase-3/9 to induce cancer cell apoptosis, inhibiting PI3K/Akt and ERK1/2 pathways, and reducing VEGF signaling	Khazdair et al. (2021)
​	Glycitin	*Glycine max (L.) Merr*	Fabaceae	Flavonoids	Affecting the function of the TOP2A protein, thereby inhibiting tumor proliferation and metastasis, and influencing its mediated cell proliferation and vasculogenic mimicry	Liu C. et al. (2025)
​	Genipin	*Gardenia jasminoides J.Ellis*	Rubiaceae	Terpenoids	Suppressing the TLR4/MyD88 signaling pathway, which consequently reduces HIF-1α-independent VEGF expression	Kim et al. (2025)
​	Angelica sinensis extract	*Angelica sinensis (Oliv.)* *Diels*	Apiaceae	Other ingredients	Restoring blood component homeostasis, enhancing vascular structural integrity, accelerating blood circulation, promoting tumor vessel normalization, and blocking pro-angiogenic factor expression and receptor interactions	Guo et al. (2025)
​	Ginkgo biloba exocarp extracts	*Ginkgo biloba L*	Ginkgoaceae	Other ingredients	Inhibiting β-catenin, VEGF, VEGFR2, and p-AKT/AKT protein expression, and VEGF and VEGFR2 mRNA, and blocking the Wnt/β-catenin-VEGF signaling pathway	Han et al. (2016)

## 2 Key bioactive components in medicinal and edible substances

Medicinal and edible substances contain diverse bioactive components, primarily composed of polysaccharides, polyphenols, flavonoids, terpenoids, and saponins as their core material basis. These components can act synergistically through multiple pathways and targets, influencing key stages in the initiation and progression of LC, demonstrating significant potential for prevention and adjunctive therapy. Consequently, further exploration of these substances holds considerable significance for the adjunctive treatment of LC and multiple other diseases.

### 2.1 Polysaccharides

Polysaccharides are macromolecules with broad biological activities, widely present in nature, and play vital physiological roles (Chakka and Zhou, 2020). In recent decades, polysaccharides isolated from various medicinal herbs have gained significant attention due to their notable bioactivities. These include anti-inflammatory (Chen G. et al., 2023), gut microbiota regulation (Wang H. et al., 2024), anti-tumor (Ju et al., 2024), antioxidant (Liu X. et al., 2025), anti-diabetic (Bo et al., 2024), and immunomodulatory effects (Tang J. et al., 2024). By inhibiting the proliferation of LC cells, natural polysaccharides can effectively restrain the disease progression. Furthermore, the modulation of gut microbiota and the amplification of immune responses contribute to the enhancement of the efficacy of conventional LC treatments (Tian et al., 2024). Overall, polysaccharides demonstrate considerable potential in LC research, offering promising avenues for developing novel clinical interventions.

The polysaccharides derived from *Laminaria japonica* (Kunbu in Chinese) serve as key active components, with research by Jin W. et al. (2025) demonstrating their significant inhibition of viability, migration, and invasion in LC cells. These effects are mediated *via* regulating the PPARG pathway, where PPARG modulates PTGS2 to reduce inflammatory mediators and suppressing the AGE-RAGE axis, where AGE-RAGE regulates HIF-1α to block angiogenesis, as identified by network pharmacology. Key downstream targets CASP3, BCL2 and CCND1 are modulated, with the S-C fraction stabilizing PPARG and interfering with CASP3 activation to balance cell apoptosis and survival. This molecular cascade from *Laminaria japonica* polysaccharides to core targets and oncogenic pathways supports their adjunctive potential in NSCLC by inhibiting cancer cell proliferation, migration, and metastasis. Although network pharmacology provides a systematic hypothetical framework for the mechanism of action of laminarin, these predicted targets and pathways still require validation through more direct molecular biology experiments, such as siRNA-mediated knockdown of key targets, to establish definitive causal relationships. *Houttuynia cordata Thunb.* Contains various bioactive compounds, among which polysaccharide HCA4S1 has shown particular relevance for LC by upregulating cleaved caspase-3 and cyclin B1 expression, suggesting induction of cell cycle arrest and apoptosis in A549 cells (Han et al., 2018). Additionally, polysaccharide Suc40 extracted from *Poria cocos (Schw.) Wolf* (Fuling in Chinese) exhibits both anti-inflammatory and anti-cancer properties, inhibiting IL-6 and TNF-α while suppressing AKT/p38 signaling pathways, and exerting anti-proliferative effects against NSCLC cells *via* activation of caspase-3/7/8/9-mediated apoptosis (Qiu et al., 2024). Similarly, network pharmacology studies have identified that GQZ lipopolysaccharide (*Lycium barbarum L.*) targets NSCLC primarily through lipopolysaccharide-related mechanisms and suppression of the PI3K/AKT1 pathway, thereby inhibiting proliferation *via* downregulation of C-MYC and PCNA and inducing apoptosis through modulation of BCL2/BAX ratio and caspase activation, as demonstrated in both *in vitro* and *in vivo* models (Zhang et al., 2022).

Collectively, these polysaccharides of medicinal and edible substances exert anti-lung cancer effects not through single-target actions but *via* multi-target mechanisms that simultaneously engage tumor cells, immune regulation, and the systemic microenvironment, forming an integrated therapeutic network.

### 2.2 Polyphenols

Polyphenolic compounds are plant-derived secondary metabolites characterized by benzene rings with multiple phenolic hydroxyl groups. They are abundantly present in common dietary sources such as fruits, vegetables, and grains, as well as in various medicinal and edible substances (Fu et al., 2024). Studies have confirmed that these compounds demonstrate a range of biological properties, including sleep regulation (Pérez-Jiménez et al., 2023), modulation of gut microbiota (Rodríguez-Daza and de Vos, 2022), anti-inflammatory (Zamani-Garmsiri et al., 2022), antioxidant (Zhong et al., 2025), antibacterial (Turuvekere Vittala Murthy et al., 2021), and immunomodulatory effects in oncology (Wang Q. et al., 2022). Through these synergistic interactions, polyphenolic compounds exert a significant influence in the field of LC research.

Curcumin, the primary active constituent of turmeric (*Curcuma longa L.*), represents an extensively studied natural compound with significant therapeutic potential, predominantly attributed to its polyphenolic nature. Research by Cho et al. (2024) confirmed that curcumin exhibits notable anti-cancer activity, particularly when synergized with piperine from black pepper. This observed synergy underscores the importance of investigating medicinal and edible substances combinations through a multi-target perspective, as advocated by network pharmacology. Polyphenols isolated from *Lonicera japonica Thunb.* demonstrate substantial anti-lung cancer properties by modulating mitochondrial-dependent apoptotic pathways in A549 cells, characterized by AKT dephosphorylation-induced Bax/Bcl-xL ratio increase, leading to caspase-9-mediated caspase-3 activation and PARP cleavage (Park et al., 2017). Similarly, cannabidiol derived from *Cannabis sativa L.*, especially cannabidiol (CBD), suppresses viability and invasion in A549 and H460 LC lines in a CB1/CB2/TRPV1-dependent manner while reducing PAI-1 expression and inducing apoptosis *via* upregulation of COX-2 and PPAR-γ (Nigro et al., 2021; Ramer et al., 2010). Furthermore, eugenol, which mediates its effects on LC by targeting β-catenin to inhibit its nuclear transport, induce cytoplasmic degradation *via* N-terminal Ser37 phosphorylation, downregulatees CSC markers, enhances apoptosis, and suppresses cell proliferation (Choudhury et al., 2021).

Together, these polyphenolic compounds act through common mechanisms against LC, ranging from apoptosis induction and proliferation suppression to signaling pathway modulation, likely due to their phenolic hydroxyl groups that facilitate interactions with key biological macromolecules.

### 2.3 Flavonoids

Flavonoids are a class of phytochemicals with diverse biological activities that contribute significantly to human health. They can effectively neutralize free radicals, making them valuable in supporting the management of various diseases. Moreover, they find extensive applications in nutraceuticals, cosmeceuticals, and pharmaceuticals (Billowria et al., 2024; Mohan et al., 2025). These compounds interact with multiple biological targets, exhibiting a range of effects including anti-inflammatory (Wang et al., 2021), antioxidant, antibacterial (Jomova et al., 2025), and notably anti-cancer properties (Jeong et al., 2023). Research indicates that flavonoids play an important role in influencing the progression of LC.

Research demonstrates that flavonoids significantly influence LC progression. *Nelumbo nucifera Gaertn.* flavonoids (LLF) induce apoptosis in A549 and H446 cells by initiating intracellular reactive oxygen species (ROS) burst, which in turn activates the p38 MAPK pathway. The activated p-p38 MAPK promotes the mitochondrial apoptotic pathway, evidenced by an increased Bax/Bcl-2 ratio, cytochrome c release, and subsequent activation of the caspase-9/3 cascade (Jia et al., 2021). Similarly, puerarin 6″-O-xyloside (PXY) suppresses the self-renewal and invasion capabilities of LC stem-like cells by inhibiting Akt/c-Myc signaling activation, thereby downregulating stem cell markers CD44, CD133, and ALDH1 (Tao et al., 2020). Components like quercetin and kaempferol regulate apoptotic factors, downregulate Bcl-2, upregulate Bax, activate caspase-3/9 to induce cancer cell apoptosis. Their activity is mediated through the inhibition of upstream pathways, including quercetin’s suppression of the PI3K/Akt/mTOR axis and genistein’s inhibition of ERK1/2 and PI3K/Akt, which collectively reduce VEGF signaling and suppress cancer stem cell characteristics. Additionally, genistein promotes autophagy *via* Bcl-xL/Beclin-1 dissociation, underscoring a multi-targeted mechanism (Khazdair et al., 2021). In particular, quercetin is frequently identified in network pharmacology studies as a key hub node, capable of simultaneously interacting with multiple targets across different pathways, which exemplifies the network-based polypharmacology of flavonoids. It should be noted that the multi-target modulatory effects of quercetin on the PI3K/Akt and autophagy pathways observed *in vitro* were predominantly achieved at relatively high concentrations. Whether equivalent effective concentrations can be attained *in vivo*, and whether such multi-target engagement may lead to off-target toxicity in normal cells, remain subjects requiring further investigation. Furthermore, linarin from *Chrysanthemum* × *morifolium (Ramat.) Hemsl.* suppresses radiation-induced cell migration and invasion in A549 cells through the inhibition of IKK-mediated IκB-α phosphorylation, which prevents NF-κB nuclear translocation, leading to the downregulation of MMP-9 expression, thereby inhibiting cancer invasion (Jung et al., 2019).

Through the coordinated regulation of key protease systems and signaling networks, these flavonoids disrupt pivotal pathways such as p38 MAPK, Akt/c-Myc, and NF-κB. This multi-target mechanism, often revealed by network pharmacology predictions, underpins their broad chemopreventive potential across the pathogenesis of LC, which includes inducing apoptosis, suppressing stemness, and limiting metastasis.

### 2.4 Terpenoids

Terpenoids are important naturally occurring bioactive substances widely found in plants and fungi. They have been demonstrated to have marked therapeutic efficacy, contributing to lowering blood glucose (Wang M. et al., 2024) and protecting liver function (Hao et al., 2024), alongside possessing anti-inflammatory (Chen J. J. et al., 2023), antioxidant (Huang et al., 2022), anti-fatigue (Zhang Y. et al., 2024), antiviral (Wimmerová et al., 2023), and crucially anticancer properties (Aly et al., 2024). Research has identified terpenoids as multi-target plant metabolites that can effectively inhibit tumor cell proliferation, migration, and invasion. They represent one of the most significant sources of bioactive compounds capable of suppressing LC progression (Markov et al., 2017).

Because of the diverse mechanisms of terpenoids, these compounds demonstrate significant potential in LC management. β-sitosterol from *Raphanus sativus L.* sprouts inhibits miR-181a-3p to upregulate the expression of its downstream target SHQ1, activates the SHQ1/UPR signaling pathway, and thereby promotes apoptosis and suppresses proliferation of LC cells. This intricate microRNA-mRNA-pathway cascade highlights the multi-layered regulatory networks that can be deciphered through integrated pharmacological approaches (Wang H. et al., 2024). Glycyrrhetinic acid (GA), a primary bioactive constituent of licorice (*Glycyrrhiza uralensis Fisch.*). Guo et al. (2024) employed an integrated strategy combining activity-based protein profiling with proteomics and histopathological validation to investigate the potential targets of GA against NSCLC, providing a multi-dimensional view of its mechanism. *In vitro* and *in vivo* results indicated GA significantly inhibited NSCLC *via* promotion of peroxiredoxin-6 and caspase-3-mediated mitochondrial apoptosis. Chen et al. (2015) conducted a study investigating that crocin from *Crocus sativus L.* suppresses proliferation in A549 and SPC-A1 cells in a dose-dependent manner, accompanied by increased p53 and decreased Bcl-2 mRNA levels, leading to apoptosis. Additionally, maslinic acid promotes apoptosis in A549 cells by modulating IAP family proteins (Bai et al., 2016; Yu et al., 2021), and the extract from *Cornus officinalis Siebold & Zucc.* shows inhibitory activity against A-549 LC cells (Li et al., 2015).

Collectively, terpenoids achieve multi-target suppression of LC proliferation by coordinately inducing apoptosis, arresting the cell cycle at G0/G1 phase, and regulating core signaling pathways including p53 and PI3K/Akt.

### 2.5 Saponins

Saponins are a diverse group of compounds widely distributed in the plant kingdom, characterized by their amphiphilic nature resulting from a hydrophobic aglycone linked to hydrophilic sugar chains *via* glycosidic bonds (Güçlü-Ustündağ and Mazza, 2007; Liu H. et al., 2025). Studies indicate that saponins demonstrate anti-tumor efficacy across various cancer types, including breast cancer (Wu et al., 2025), colorectal cancer (Bai et al., 2025), and osteosarcoma (Jin K. et al., 2025). Recent studies have revealed that saponins derived from medicinal and edible substances also demonstrate promising bioactivity in LC management, showing potential for therapeutic application.

Saponins exhibit multi-target anti-lung cancer effects through coordinated mechanisms. Lily saponin, in addition to its significant anti-hepatocellular carcinoma effects (He et al., 2024). It also has been demonstrated to have potential effects on human LC cell proliferation, apoptosis, migration, and invasion. For example, steroidal glycoside six can exhibit potent cytotoxicity against the A549 lung cancer cell line, with a half-maximal inhibitory concentration of 1.49 μM (Zhou et al., 2024; Luo et al., 2018). Furostanol saponin, a novel compound isolated from *Asparagu cochinchinensis (Lour.) Merr.,* significantly inhibits H1299 LC cell proliferation and induces apoptosis, by doing so, it slows disease progression (Zhang R.S. et al., 2021). This anti-lung cancer activity of steroidal saponins provides a modern research example of TCM against tumors. Furthermore, studies have identified two new furostanol saponins, macrostemonoside E and F, as key active components responsible for the anti-lung cancer activity in *Allium chinense G.Don* (Wang Y. et al., 2019). Ophiopogonin B has been shown to inhibit cell viability and proliferation, consequently suppressing invasion and migration in NSCLC cell lines through mechanisms associated with epithelial-mesenchymal transition (EMT) (Zhang S. et al., 2021). A recent study by Feng L. et al. (2024) confirmed that ginsenoside Rb1 induces LC cell apoptosis by altering protein levels and activating the external apoptotic pathway. Additionally, Jujuboside B triggers endoplasmic reticulum stress, leading to the activation of the PERK-eIF2α-ATF4 signaling axis. This cascade results in the upregulation of ATF3, which subsequently suppresses the expression of SLC7A11. The downregulation of SLC7A11 depletes glutathione and inactivates Gpx4, culminating in lethal lipid peroxidation and ferroptosis (Kim and Ko, 2025). These saponins collectively exert multi-target anti-tumor effects by synergistically regulating the Bcl-2/Bax ratio, activating the caspase cascade to induce apoptosis, while simultaneously inhibiting EMT and matrix metalloproteinase (MMP) activity, which in turn suppresses LC cell proliferation, migration, and invasion.

Together, these saponins exert their anti-tumor effects by coordinately regulating the Bcl-2/Bax ratio, activating caspase cascades to induce apoptosis, while simultaneously inhibiting EMT and MMPs activity, thereby ultimately suppressing LC cell proliferation, migration, and invasion.

### 2.6 Other bioactive ingredients

Bioactive components encompass a diverse range beyond the previously mentioned polysaccharides, flavonoids, polyphenols, terpenoids, and saponins. Other notable constituents include lectins, lipids, and alkaloids. Various bioactive components from medicinal and edible substances demonstrate anti-lung cancer effects through distinct mechanisms. Seabuckthorn Pulp Oil combined with DTX showed synergistic anti-cancer activity through caspase-independent autophagy and senescence induction, accompanied by increased ROS production, LC3 protein expression, G1 phase arrest, and enhanced senescence-associated β-galactosidase activity (Batbold and Liu, 2022). *Polygonatum cyrtonema* lectin induces significant anti-tumor effects through cell death and autophagy, with ROS scavenger NAC inhibiting PCL-induced cell death and autophagy in A549 cells (Liu et al., 2016). Neferine, an alkaloid from *N. nucifera Gaertn.*, induces apoptosis in NSCLC cells by elevating ROS and reducing the BCL2/BAX ratio. Simultaneously, it suppresses invasion and EMT by targeting ROCK1, inhibiting MLC phosphorylation, and reversing EMT markers. This dual action effectively blocks tumor growth and metastasis. (Hu et al., 2023). Cinnamon essential oil triggers cell death in LC cells by causing cell cycle arrest, increasing ROS accumulation, inducing mitochondrial depolarization, and elevating caspase-3, 8, and nine levels (Mohanty et al., 2024). Abeesh et al. (2021) demonstrated that sword bean extract inhibits cell growth, induces apoptosis, and significantly suppresses the development of mouse ascites and solid tumors. Echinacoside from *Cistanche deserticola Ma* induces pyroptosis in NSCLC cells through the mitochondrial-mediated Raf/MEK/ERK signaling pathway (Tang Y. et al., 2024). Coixol and a known compound isolated from coix bran show anti-proliferative effects against NSCLC cells (Lee et al., 2008; Wang L. H. et al., 2024).

In summary, medicinal and edible substances demonstrate unique advantages and potential in LC prevention and treatment through their rich bioactive components and multi-component, multi-target synergistic mechanisms. Therefore, future research should focus on elucidating the multi-target synergistic mechanisms of active ingredients such as polysaccharides, polyphenols, and flavonoids in medicinal and edible substances, along with their specific molecular mechanisms in intervening key LC signaling pathways.

## 3 Mechanisms of action of medicinal and edible substances on lung cancer

Medicinal and edible substances exert significant inhibitory effects on the development of LC through a multidimensional and hierarchically progressive synergistic mechanism. Insights from network pharmacology analyses suggest that the diverse bioactive components in medicinal and edible substances converge on a core network of signaling pathways related to inflammation, oxidative stress, immunity, and cell proliferation. The primary sequence of actions is as follows: (1) Targeted suppression of the inflammatory initiation phase. Medicinal and edible substances exhibits potent anti-inflammatory activity (Qu et al., 2023), which can block the activation of specific signaling pathways, downregulate the release of pro-inflammatory cytokines, and reduce the infiltration and activation of inflammatory cells, thereby inhibiting inflammation-mediated malignant transformation of LC. (2) Efficient clearance of oxidative stress products to further alleviate inflammatory damage. Signaling through inflammasomes and Toll-like receptors activates inflammatory responses and promotes excessive generation of ROS and reactive nitrogen species, initiating oxidative stress (Ramos-González, et al., 2024). Under conditions of inflammatory stress, medicinal and edible substances exert their protective effect primarily through their potent antioxidant capacity. First, they specifically scavenge excess intracellular ROS. This direct ROS reduction mitigates key downstream consequences, including DNA damage, telomere shortening, and mitochondrial dysfunction. Furthermore, by limiting ROS levels, medicinal and edible substances interrupts the positive feedback loop between inflammatory activation and oxidative stress. Consequently, this comprehensive action ultimately leads to reduced inflammatory injury. (3) Remodeling anti-tumor immune responses and reversing the immunosuppressive microenvironment. Oxidative stress exerts a dual influence on tumor immunity: it can promote the initiation of immune responses while also impairing their sustainability. Moderate oxidative stress may induce tumor cell apoptosis, enhance the tumoricidal capacity of immune cells, and increase the presentation of tumor epitopes, thereby potentiating tumor immunotherapy. In contrast, high levels of oxidative stress are often associated with tumor growth and metastasis and may compromise the efficacy of immunotherapeutic interventions (Huang et al., 2023; Zhang H. et al., 2023). To counter the immune imbalance induced by oxidative stress, medicinal and edible substances promote dendritic cell maturation and antigen-presenting capacity, and enhance the proliferation and cytotoxic activity of T cells, thus exerting immunomodulatory effects. (4) Inhibition of tumor angiogenesis. Immune regulation is closely linked to tumor angiogenesis. Immune cells participate in the production and release of various pro-angiogenic or anti-angiogenic factors, thereby modulating tumor vascular formation and the proliferation, migration, and activation of endothelial cells (Kim et al., 2022). Studies indicate that abnormal tumor vasculature is a critical factor influencing cancer progression, the development of therapy resistance, and patient prognosis, partly by impairing the function of effector immune cells within the tumor microenvironment (TME) (De Palma et al., 2005; Coffelt et al., 2011). (5) Multi-pathway regulation of cell fate decisions. Factors such as VEGF act on tumor cell surface receptors, activating signaling pathways like MAPK/ERK and PI3K/Akt, directly promoting tumor cell proliferation and survival. This establishes a positive feedback loop of “angiogenesis–tumor growth,” which is a key mechanism underlying sustained tumor progression (Lin et al., 2017; Goggins et al., 2023).

**FIGURE 2:**
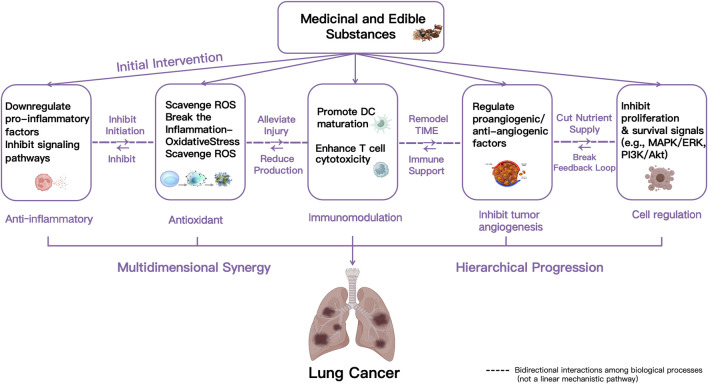
Schematic diagram of the mechanisms of action of medicinal and edible substances on LC.

In summary, the inhibitory effect of medicinal and edible substances on LC is mediated by a synergistic cascade of mechanisms, rather than isolated actions. This multi-faceted engagement sequentially targets key pathophysiological processes, including inflammation, oxidative stress, immune dysregulation, angiogenesis, and cell fate. As schematically represented in Figure 2, these interconnected stages work synergistically to enhance the overall therapeutic efficacy. The following sections will detail the roles of specific medicinal and edible substances in mediating anti-inflammation, antioxidation, immunomodulation, inhibition of tumor angiogenesis, and cellular regulation.

### 3.1 Anti-inflammatory effects

Inflammation represents the body’s normal immune response to invasive pathogens. Chronic inflammation is involved in multiple stages of cancer progression, including tumor initiation, cellular transformation, invasion, angiogenesis, proliferation, and metastasis (Zhao et al., 2021). Research indicates that chronic inflammation, which often accompanies and stimulates cancer development, contributes significantly to the high mortality rate in LC. Key mediators such as NF-κB, transcription activators, signal transducers, and hypoxia-inducible factors serve to bridge inflammation and cancer. Furthermore, driven by these complex factors, the inflammatory microenvironment promotes LC development through mechanisms such as inducing EMT in tumor cells (Odarenko et al., 2023). Various medicinal and edible substances, including flavonoids and polyphenols, exert influence on LC progression through their anti-inflammatory properties.

Anethole and shikimic acid, polyphenolic compounds derived from *Illicium verum Hook. f.*, have been reported through multiple mechanisms, including anti-inflammatory and antioxidant activities, to prevent and treat LC. Their anti-inflammatory effects are primarily demonstrated by inhibiting the NF-κB pathway to downregulate downstream TNF-α and fibronectin, and suppress oxidative stress-induced p53 overexpression. Meanwhile, they scavenge ROS, reduce MDA levels, and alleviate lung cell oxidative damage, disrupting the inflammatory-oxidative loop in TME to hinder LC progression (Abdelaziz et al., 2024). Menthol, a terpenoid from *Mentha haplocalyx Briq.* (Bohe in Chinese), exhibits anticancer activity against various human cancers including LC *via* regulating MAPK and PI3K/Akt pathways, inducing cell death, inhibiting invasion and migration, causing cell cycle arrest, upregulating Bax and p53 genes, and remodeling the chronic inflammatory microenvironment by modulating inflammatory mediators like TNF, IL-6, IFN-γ, and IL-8 (Tafrihi et al., 2021). Catalpol, a terpenoid from *Rehmannia glutinosa (Gaertn.) Libosch. ex DC.*, significantly inhibits TGF-β1-induced migration and invasion in A549 cells through blocking Smad2/3 and NF-κB signaling activation, leading to suppression of inflammation-driven LC progression (Wang Z. et al., 2019; Laurindo et al., 2025). Similarly, amygdalin (Lin et al., 2022) and *Polygonatum odoratum* lectin (Li et al., 2014) also inhibit LC development through NF-κB pathway-mediated anti-inflammatory mechanisms. Dioscin, a natural saponin, reduces LC cell proliferation, invasion, and migration by decreasing p-AKT, MMP2, and PCNA expression, consequently inhibiting lung nodule formation, lung injury, and mortality in mouse models (Xi et al., 2022). Perilla seed oil, rich in α-linolenic acid, exerts anti-inflammatory and antioxidant effects by reducing TNF-α-induced ROS, inhibiting JNK phosphorylation and FOXO1 expression to lower MnSOD levels for antioxidant effects. It also suppresses NF-κB activation, decreasing downstream pro-inflammatory mediators, including IL-1β, IL-6, IL-8, TNF-α, COX-2, in A549 cells. These upstream links involving ROS and JNK and downstream links extending to FOXO1, MnSOD, NF-κB, and various cytokines clarify its anti-inflammatory and antioxidant mechanisms. (Tantipaiboonwong et al., 2021). Piperlongumine, an alkaloid from *Piper longum L.*, induces apoptosis and inhibits invasion and metastasis in LC cells, while significantly reducing tumor volume and M2 macrophage polarization through endoplasmic reticulum stress induction in co-culture systems (Zhou et al., 2025).

The above study reveals that medicinal and edible substances exert their effects by inhibiting classic inflammatory pathways such as JNK and NF-κB. However, the underlying mechanisms of chronic inflammation-induced carcinogenesis lie in its ability to induce sustained activation of a complex signaling network, driving malignant remodeling of tissue structure. This process shares common characteristics across multiple chronic inflammatory diseases. For example, in chronic rhinosinusitis, *Staphylococcus aureus* infection upregulates MUC13 and synergistically activates the MEK1/2 and WNT2B signaling axis, leading to persistent mucosal inflammation and abnormal tissue remodeling (Feng et al., 2025b). In the development of LC, a similar logic applies: chronic inflammatory signals in the tumor microenvironment such as TGF-β and TNF-α induce epithelial-mesenchymal transition, promote extracellular matrix degradation, and ultimately drive invasion and metastasis of cancer cells by activating evolutionarily conserved pathways including MAPK/ERK, the downstream of MEK, and Wnt/β-catenin.

It is worth emphasizing that the anti-inflammatory effects of medicinal and edible substances precisely target this integrated signaling network. For instance, Catalpol mentioned in this section can inhibit TGF-β1-induced migration and invasion of A549 cells by blocking Smad2/3 and NF-κB signaling; Curcumin and Anethole have also been widely reported to inhibit the Wnt/β-catenin pathway. These findings indicate that medicinal and edible substances not only suppress inflammatory factors as per traditional understanding but also reprogram the signaling landscape—by regulating core hub pathways such as MEK/ERK and Wnt, they block the malignant transformation process from inflammation to epithelial-mesenchymal transition and tissue remodeling at the upstream level, thereby intervening in the progression of LC at a deeper level.

### 3.2 Antioxidant effects

Redox reactions regulate fundamental biochemical processes, including energy production, substance metabolism, respiration, detoxification, and signal transduction. Due to their sustained proliferation, tumor cells maintain heightened metabolic activity and generate elevated levels of ROS compared to normal cells (Hayashi et al., 2024). ROS, encompassing both free radicals and non-radical species, serve as central mediators of oxidative stress, damaging cellular components such as lipids, proteins, and DNA, while also disrupting pulmonary homeostasis to create a microenvironment conducive to tumor development (Pizzino et al., 2017; Smolarz et al., 2025). The initiation and progression of LC are directly linked to systemic redox imbalance (Jaruga et al., 1994; Zabłocka-Słowińska et al., 2018; Miao et al., 2022), making antioxidant intervention an important aspect of its prevention and treatment.

An *in vitro* study demonstrated that raspberry seed methanol extract selectively inhibits the growth of LC A-549 cells while remaining non-toxic to normal lung cells. This extract effectively scavenges hydroxyl and superoxide anion radicals closely associated with LC pathology, indicating its anti-cancer effect is linked to potent antioxidant properties (Simonovic et al., 2021). Longan Pericarp-derived Phenolics extract demonstrated free radical scavenging capacity against DPPH and hydroxyl radicals, along with ferrous ion chelating ability in antioxidant assays (Bai et al., 2019). Research by Srisomsap et al. (2024) found that mulberry leaves (*Morus alba L.*) contain various bioactive polyphenolic compounds, particularly chlorogenic acid (CGA), which selectively inhibits the growth of MCF-7 cells and exhibits effective free radical scavenging activity against DPPH radicals, thereby suppressing migration and invasion activities against A549 LC cells. Hesperidin downregulates MMPs expression and enhances antioxidant status to counter nicotine toxicity and inhibit smoking-induced LC. Its antioxidant capacity also suppresses tumor cell proliferation in benzopyrene-induced LC mouse models, while additionally inhibiting NSCLC cell proliferation and promoting cell death through the miR-132/ZEB2 signaling pathway (Balakrishnan and Menon, 2007; Kamaraj et al., 2009; Tan et al., 2020). Ononin, a flavonoid extracted from *Astragalus mongholicus Bunge*, binds to HIF-1α through hydrogen bonds with TYR-131, GLN-133, GLN-134 and hydrophobic interaction with LEU-172, and when combined with radiotherapy, it suppresses the HIF-1α to VEGF pathway overactivation. This regulatory process inhibits LC cell growth by reducing proliferation and induces apoptosis *via* increasing Bax expression, decreasing Bcl-2 expression and elevating Cleaved Caspase3 level *in vitro*. *In vivo* studies using mouse models show this combination reduces lung tumor size and proliferation, promotes cancer cell apoptosis, alleviates abnormal HIF-1α pathway activation, and protects liver function by maintaining albumin and alkaline phosphatase homeostasis (Zhang Y. M. et al., 2024). Purslane (*Portulaca oleracea L.*) and its main active components influence LC development through multiple mechanisms, including antioxidant effects, inhibition of tumor angiogenesis, and suppression of LC cell proliferation. Its seed oil, whole plant chloroform extract, and alkaloids concentration-dependently inhibit LC cell proliferation and reduce cell viability. Lee et al. (2021) confirmed that FDY2004, an anti-cancer herbal formulation composed of *Moutan Cortex, Persicae Semen, and Rhei Radix et Rhizoma,* affects LC through molecular mechanisms involving the regulation of cell proliferation and growth, cell survival and death, as well as oxidative stress responses.

### 3.3 Immunomodulation effects

The immune response plays a vital role in defending against external antigens while maintaining tolerance to self-antigens (Han et al., 2025). This balance, known as immune homeostasis, is essential for overall health. When tumor cells invade and proliferate, immune cells such as macrophages and T cells are activated by signaling molecules within TME, mounting a defensive response against LC cells. Consequently, immunomodulation plays a crucial role in influencing the progression and prognosis of LC.

CAVAPs from *Citrus maxima (Burm.) Merr.* exhibit multiple biological activities. They demonstrate significant DPPH free radical scavenging capacity in terms of antioxidant activity, and show cytotoxic effects against both human breast cancer MCF-7 cells and LC HCC827 cells. In Addition, they possess immunoenhancing properties. Research by Shen et al. (2017b) found that CAVAPs may activate macrophages through the MAPK and NF-κB signaling pathways. This is evidenced by the increased phosphorylation of ERK, JNK, p38 and p65 proteins, leading to the upregulated expression and secretion of key immune mediators including iNOS, TNF-α, IL-1β and IL-6. Similarly, another study revealed that CAVAPs exhibit stronger DPPH scavenging activity, FRAP, and reducing power, influencing LC mediated by antioxidant mechanisms (Shen et al., 2017a). These findings indicate CAVAPs can affect LC development through dual mechanisms of antioxidant activity and immunoregulation. 6-Gingerol, a bioactive polyphenolic compound extracted from fresh or dried ginger, demonstrates broad-spectrum anticancer activity. Kang et al. (2023) discovered that 6-gingerol inhibits LC cell proliferation and induces DNA damage response, cell cycle arrest, and apoptosis in NSCLC cells. Furthermore, iron metabolism enhances anticancer capacity by maintaining iron homeostasis through PD-L1/Akt/β-catenin/WIP signaling axis regulation. Specifically, PD-L1 activates PI3K/Akt to inhibit β-catenin degradation, promoting β-catenin binding to the WIP promoter and subsequent WIP transcription. This axis drives LC cell proliferation, migration, and invasion, while iron metabolism-mediated PD-L1 downregulation disrupts this pro-tumorigenic cascade. Additionally, 6-gingerol can modulate PD-L1 expression, playing a vital role in cancer immunotherapy (Yu et al., 2020). Kang et al. (2021) experimentally tested the effects of the total flavonoids from *Taraxacum mongolicum Hand.-Mazz.* on cancer cells in LC models, finding increased levels of CD4^+^, CD8^+^ T cells and CD4^+^/CD8^+^ ratio, elevated IL-2, IL-3, IFN-γ, and TNF-α levels, along with significantly decreased Ki67 expression, thus inhibiting LC cell proliferation and enhancing host immunity. Platycodin D, the main pharmacological component of *Platycodon grandiflorus (Jacq.) A.DC*., demonstrates various pharmacological activities through oxidative stress defense mechanisms. Studies have confirmed that Platycodin D exhibits potential anti-tumor, anti-cachexia, and immunomodulatory activities in athymic nude mice bearing lung H520 tumor cells (Park et al., 2014; Feng M. et al., 2024), likely combating LC exacerbation through immune enhancement. Moracin N (*M. alba L. leaves*) directly binds to the E158 residue of PD-L1, triggering PD-L1 tyrosine phosphorylation to promote its degradation. This binding disrupts the PD-L1/PD-1 interaction, reversing CD8^+^ T cell exhaustion by reducing CD8^+^PD-1^+^ subsets and enhancing CD8^+^GZMB^+^ cytotoxic T cell function. Together with synergistic effects from anti-PD-1 antibodies, MAN modulates the tumor immune microenvironment, ultimately suppressing LC tumorigenesis, progression, and metastasis (Ye et al., 2024).

In addition to the aforementioned strategies that directly target immune checkpoints or activate effector immune cells, intervening in the source of production of suppressive elements in the tumor immune microenvironment is another fundamental regulatory approach. Among these, Cellular Senescence and its accompanying senescence-associated secretory phenotype, a state characterized by the massive secretion of inflammatory factors, chemokines and proteases, has been identified as a key driver of shaping the immunosuppressive tumor immune microenvironment in recent years. Studies have confirmed that the abnormal expression of senescence-associated genes in tumor tissues is closely associated with poor prognosis. The underlying mechanism is that the inflammaging microenvironment created by senescent cells can directly induce T cell exhaustion and promote the infiltration of regulatory T cells, thereby facilitating tumor immune evasion, recurrence and metastasis. For instance, in head and neck squamous cell carcinoma, the specific expression profile of cellular senescence-related genes has been verified as an independent risk factor for predicting tumor recurrence and patient survival, which clearly reveals the causal link among senescence, immune dysregulation and tumor progression (Feng M. et al., 2025). Studies indicate that medicinal and edible substances can modulate cellular senescence-associated pathways, thereby ameliorating the tumor immune microenvironment and exerting potential effects in the prevention and treatment of LC. For example, Quercetin, as mentioned earlier, not only exhibits antioxidant properties and inhibits pro-cancer pathways such as PI3K/Akt, but also induces pro-apoptotic autophagy in LC cells *via* the SIRT1/AMPK signaling pathway. SIRT1 is a core molecule in the classical anti-aging pathway, and its activation can directly delay cellular senescence and reduce DNA damage. The upregulation of SIRT1 by Quercetin serves as the key link connecting “antioxidation-anti-aging-anti-lung cancer”at the molecular level. Consequently, medicinal and edible substances agents show promise in LC prevention and therapy by targeting senescence-associated signaling to favorably remodel the immunosuppressive tumor microenvironment.

### 3.4 Inhibit tumor angiogenesis effects

Tumor angiogenesis refers to the formation of abnormal vascular networks within tumors, resulting from an imbalance between pro-angiogenic and anti-angiogenic signals (Alsaab et al., 2021). Vascular endothelial growth factors (VEGFs) and their receptors (VEGFRs) play a central role in this process. The VEGF pathway, a key mediator of angiogenesis, has become an attractive target in multiple malignancies, including LC. Studies show that tumor angiogenesis not only supplies oxygen and nutrients required for LC cell proliferation but also establishes pathways that promote metastasis and create hypoxic regions, thereby contributing to drug resistance (Yan and Shi, 2024).

*Ganoderma lucidum (Curtis) P. Karst.* (Lingzhi in Chinese), a widely used medicinal food, demonstrates effective anti-tumor activity in EGFR-mutant NSCLC by downregulating EGFR expression through proteasomal and lysosomal degradation mediated by its bioactive components such as WSG and rLZ-8. Firstly, it suppresses cell survival and tumor progression by blocking the EGFR-mediated activation of the PI3K/Akt/mTOR and ERK/AP-1 cascades. Secondly, it attenuates the Wnt/β-catenin pathway through inhibition of LRP6 phosphorylation, thereby restraining EMT and angiogenesis. Additionally, it modulates the tumor microenvironment by activating M1 macrophages and NK cells while reducing immunosuppressive cytokines like IL-10 and TGF-β, thereby enhancing anti-tumor immunity and overcoming EGFR-TKI resistance (Zhang et al., 2025). Glycitin, a flavonoid extracted from *Glycine* max *(L.) Merr.* (Dandouchi in Chinese), shows potential for treating NSCLC. It exhibits significant anti-tumor activity by inhibiting tumor proliferation and metastasis through affecting the TOP2A protein function. A significant positive correlation exists between TOP2A expression levels and the expression of vasculogenic mimicry-related factors, suggesting that glycitin may exert therapeutic effects on NSCLC by targeting TOP2A and influencing its mediated cell proliferation and vasculogenic mimicry (Liu C. et al., 2025). Genipin from *Gardenia jasminoides J.Ellis* affects LC progression by directly suppressing the EGFR/JAK1/STAT3 signaling pathway. It binds to EGFR extracellular domain III to interfere with EGF binding and block EGFR phosphorylation then further inhibits downstream JAK1/STAT3 phosphorylation. This also modulates EMT-related proteins by upregulating E-cadherin and downregulating N-cadherin MMP9 Snail and RhoA. It induces G1/S phase arrest and apoptosis of LC cells and attenuates their migration and invasion a mechanism distinct from genipin’s UCP2-dependent regulation (Kim et al., 2025). A study conducted by Guo et al. (2025) has demonstrated that angelica sinensis extract by restoring blood component homeostasis, enhancing vascular structural integrity, accelerating blood circulation, promoting tumor vessel normalization, and blocking pro-angiogenic factor expression and receptor interactions improve tumor microenvironment hypoxia, inhibit aberrant angiogenesis, and delay progression from pulmonary nodules to LC. Han et al. (2016) found that *Ginkgo biloba L.* exocarp extracts influence LC development primarily by inhibiting tumor angiogenesis, as evidenced by reduced CD34 expression and suppressed microvessel density. They downregulate upstream Wnt3a to block β-catenin, further suppressing downstream VEGF and VEGFR2 transcription and subsequent AKT phosphorylation in LLC transplanted C57BL/6 mouse tumors. This Wnt/β-catenin-VEGF-VEGFR2-AKT pathway modulation is confirmed by dose-dependent inhibition of β-catenin, VEGF, VEGFR2, p-AKT/AKT protein expression and VEGF, VEGFR2 mRNA levels.

In summary, tumor angiogenesis serves as a critical process in LC tumor growth and metastatic dissemination, playing an important role in promoting LC cell metastasis, proliferation, and spread. Medicinal and edible substances represented by glycitin and genipin can influence LC progression by inhibiting angiogenesis. Therefore, the suppression of angiogenesis has been recognized as an attractive target for anti-lung cancer therapy.

### 3.5 Cell regulation effects

Cellular regulation refers to the molecular mechanisms through which cells control their own proliferation, differentiation, apoptosis, and metabolism to maintain internal stability and normal physiological functions. When this regulation becomes dysregulated in cancer, cells gain the ability to proliferate indefinitely and evade cell death, leading to tumor development. Two critical aspects of this process are the evasion of apoptosis and the sustained activation of proliferation signals. Apoptosis, a classic programmed cell death mechanism, is normally initiated to eliminate damaged cells and maintain homeostasis in response to stimuli such as inflammation or oxidative stress (Mohammad et al., 2015; Vaghari-Tabari et al., 2021). Simultaneously, uncontrolled proliferation signals drive continuous tumor expansion. Therefore, restoring normal cellular control through the reactivation of apoptosis or suppression of proliferation constitutes a fundamental therapeutic approach in cancer management.

*Phyllanthus emblica L.* extract enhances its anti-lung cancer efficacy through green-synthesized iron oxide nanoparticles, where polyphenolic components stabilize particles *via* hydroxyl groups and generate reactive oxygen species that exacerbate DNA damage and promote cancer cell apoptosis, demonstrating superior performance compared to uncoated particles (Thoidingjam and Tiku, 2019). While this study employed advanced nanotechnology to enhance delivery efficiency, it simultaneously introduced new variables. The extent to which the observed potent anticancer efficacy is attributable to the medicinal and edible substances extract itself *versus* the nanocarrier requires discrimination through more rigorous controlled experiments. Sesamol, a polyphenolic lignan found in sesame seeds and oil, inhibits SK-LU-1 cell growth by increasing caspase-8, -9, and -3/7 activities, indicating apoptosis induction through both extrinsic and intrinsic pathways (Siriwarin and Weerapreeyakul, 2016). Additional polyphenolic compounds regulating LC cells include kaempferol from sand ginger (Zhang J. et al., 2023), myristicin from *Myristica fragrans Houtt.* (Maheswari et al., 2018), and compounds from *Euryale ferox Salisb.* (Nam et al., 2019). Quercetin, a natural flavonoid widely present in fruits and vegetables, inhibits cell viability and induces mitochondrial-dependent apoptosis in A549 and H1299 cells while promoting LC3-II and beclin-1 expression and suppressing p62, with autophagy inhibition effectively reducing quercetin-induced apoptosis (Guo et al., 2021). Mogrol, a terpenoid from *Siraitia grosvenorii (Swingle) C.Jeffrey ex A.M.Lu & Zhi Y.Zhang,* significantly suppresses proliferation and migration in multiple NSCLC cell lines (A549, H1299, H1975, SK-MES-1) and triggers excessive autophagy and autophagic flux leading to autophagic cell death (Li et al., 2022). Research by Imam et al. (2022) has shown that lactucin from *Cichorium intybus L.* inhibits lung adenocarcinoma cell proliferation without affecting normal lung cells, significantly arresting the cell cycle at G0/G1 phase and inducing apoptosis through downregulating the MAPK/ERK *via* reduced MEK/ERK phosphorylation to lower cyclin D1/CDK2/4 and upregulate p53-p21, arresting cell cycle at G0/G1. It induces apoptosis by p53-Bax upregulation and Bcl-2 suppression, and activates PTEN to inhibit Akt. Additionally, it binds PGM, PKM, LDHA and PDH (central carbon metabolism enzymes) to limit glucose use and lactate production, synergistically inhibiting cancer *via* MAPK and metabolic pathway suppression. Similarly, ursolic acid targets the MEK/ERK upstream cascade, suppressing ERK phosphorylation in NSCLC cells. This inhibition directly attenuates the activation of downstream transcription factor CREB, thereby blocking the transcriptional expression of gelatinases MMP-2 and MMP-9. The anti-invasive activity of ursolic acid is mediated by suppressing the MEK/ERK/CREB-mediated expression of MMP-2 and MMP-9, combined with the simultaneous induction of RECK, an endogenous MMP-9 inhibitor, which together potently inhibit ECM degradation (Son and Lee, 2020). Thus, the inhibition of the MEK/ERK pathway by these medicinal and edible substances presents a stark contrast to its aberrant activation by environmental carcinogens, highlighting the significant interventional value of medicinal and edible substances. For example, in head and neck squamous cell carcinoma, nicotine, a primary component of tobacco smoke, activates the CHRNA5 receptor, upregulates carboxylesterase 1 expression, and leads to sustained activation of the MEK/ERK signaling axis, thereby driving tumor cell migration and invasion, as demonstrated in (Feng C. et al., 2024). Given that smoking is the predominant risk factor for LC this CHRNA5-CES1-MEK/ERK oncogenic axis is likely to play a similarly central role in the initiation and progression of LC. Therefore, medicinal and edible substances capable of targeting this pathway, such as ursolic acid and lactucin, not only provide a molecular basis for countering the cancer-promoting effects of nicotine and other environmental carcinogens but also establish a mechanistic foundation for their potential role as chemopreventive agents in LC management. Other medicinal and edible substances regulating LC through cellular mechanisms include lobetyolin and lobetyol (Wang M. C. et al., 2022), *Alpinia officinarum Hance* extract (Alasmary et al., 2019), and the extract of *Foeniculum vulgare Mill.* (Ke et al., 2021).

Collectively, cellular regulation represents the most prevalent mechanism by which medicinal and edible substances affect LC, primarily through inducing tumor cell apoptosis and inhibiting tumor cell proliferation, invasion, and metastasis.

## 4 Conclusion and future perspective

Through systematic review and analysis of medicinal and edible substances influencing LC, we observe that their effects are not mediated through single targets, but rather involve multi-target, multi-pathway, and multi-angular mechanisms. Such a network-based approach not only helps overcome drug resistance but also synergizes with conventional radiotherapy, chemotherapy, and targeted therapy. Additionally, medicinal and edible substances show potential in reducing treatment toxicity, improving quality of life, modulating the tumor microenvironment, and maintaining a favorable safety profile, positioning them as a promising complementary approach to Western medical treatments.

The complexity of the mechanism of medicinal and edible substances effect LC is clearly demonstrated by the pharmacological profiles of specific bioactive constituents. Taking Quercetin as an example, it not only directly inhibits cell proliferation by suppressing the PI3K/Akt/mTOR pathway but also induces autophagic cell death through activation of the SIRT1/AMPK signaling axis, while concurrently serving as a powerful antioxidant. This concurrent regulation of multiple critical cellular processes, spanning proliferation, cell death and oxidative stress, provides it with distinctive potential to overcome resistance to conventional targeted therapies, since tumor cells find it challenging to evade such a coordinated multitarget attack through mutations in isolated pathways. Moreover, glycyrrhetinic acid has been shown to promote mitochondrial apoptosis by targeting executioner caspase three and peroxiredoxin 6. In a similar manner, ginsenoside Rb1 regulates the Bcl-2/Bax balance and stimulates the extrinsic apoptosis pathway. These compelling examples substantiate the paradigm that medicinal and edible substances produce their therapeutic benefits through the coordinated regulation of multiple central signaling nodes. Nevertheless, this multi-constituent nature represents both a source of therapeutic advantage and a fundamental challenge for clinical translation. A critical limitation in current research lies in the nascent understanding of complex pharmacodynamic interactions, both among medicinal and edible substances constituents themselves and between medicinal and edible substances and conventional therapies. The enhanced anticancer activity observed with the combination of curcumin and piperine is not an isolated phenomenon; rather, it illustrates a broader principle, which components with favorable bioavailability profiles may enhance the biological efficacy of poorly permeable compounds through pharmacokinetic modulation. Alternatively, one constituent may potentiate another’s inhibitory effect on central signaling pathways by suppressing compensatory negative feedback mechanisms. Conversely, potential antagonistic interactions warrant serious consideration. For instance, when a potent pro-apoptotic agent coexists with a component that activates cell survival signaling, the net therapeutic effect may be substantially diminished. Elucidating these intricate interactions is paramount for establishing evidence-based clinical combinations that ensure both efficacy and safety.

Consequently, developing novel derived food products centered on medicinal and edible substances or promoting interdisciplinary integration with other fields represents a promising future direction. Nevertheless, this field faces significant bottlenecks in clinical translation. (1) Low Evidence Hierarchy. To ensure the reliability of the investigational compounds, we initially performed a pan-assay interference compounds (PAINS) filter on all relevant molecules to exclude potential false-positive interference. Building upon this, a systematic evaluation of the existing evidence framework was conducted. The results indicate that the vast majority of current evidence remains confined to the level of *in vitro* cellular experiments and animal models. Although these preclinical studies can elucidate preliminary pharmacological mechanisms, they fail to adequately recapitulate the complex tumor microenvironment, metabolic processes, and systemic immune responses in humans. Furthermore, there is a notable scarcity of large-scale, randomized, double-blind, placebo-controlled clinical trials to verify their efficacy and safety, with the limited available data originating only from small-scale clinical observations (a detailed summary of the compound screening and evidence grading is provided in the Supplementary Table). Consequently, direct extrapolation of effective *in vitro* concentrations to human therapeutic doses is highly uncertain, and their ultimate clinical relevance remains undetermined at present. (2) Limitations in Experimental Design. Numerous studies exhibit shortcomings in their experimental design. First, regarding dosage, some studies utilize extremely high concentrations of extracts or monomers in pursuit of significant effects, far exceeding physiologically achievable levels through normal dietary intake or safe supplementation, thereby casting doubt on their practical relevance. Second, there is an issue of model homogeneity; research is heavily concentrated on NSCLC cell lines such as A549, while studies on SCLC and other LC subtypes are scarce, failing to represent the full spectrum of LC pathologies. Third, mechanistic investigations are often incomplete; while network pharmacology predictions provide valuable multi-target insights, most lack subsequent rigorous genetic or biochemical validation, for instance through gene knockout or overexpression experiments, resulting in insufficiently robust causal links within the proposed mechanism of action pathways. (3) Lack of Research on Composition Complexity and Interactions. Medicinal and edible substances are inherently complex systems comprising multiple components. Existing research predominantly focuses on single active ingredients, overlooking the naturally occurring context of synergistic or antagonistic interactions. The synergistic effect between curcumin and piperine mentioned in this review represents merely an isolated example. In most cases, there is a significant knowledge gap regarding how multiple coexisting components interact in terms of their pharmacokinetics, encompassing absorption, distribution, metabolism, and excretion, and their pharmacodynamics. A potent pro-apoptotic component could potentially be antagonized by another component that activates survival signaling pathways, leading to an underestimation or masking of the overall therapeutic effect.

To address these challenges, future research should prioritize breakthroughs in the following areas: (1) Deepening the Scientific Understanding of Medicinal and Edible Substances. Integrate multi-omics technologies, including metabolomics and proteomics, with artificial intelligence to systematically decipher their multi-target mechanisms of action. Employ network pharmacology and other advanced methodologies to establish causal relationships between active components, key biological targets, and signaling pathways, and to explore the pharmacokinetic synergistic principles when combined with chemical drugs. (2) Investigating Inter-component Interactions within Medicinal and Edible Substances. Strengthen research focused on uncovering synergistic or antagonistic effects among different bioactive constituents in these complex mixtures, thereby providing a scientific basis for precise formulation. (3) Promoting Interdisciplinary Integration. Foster deeper collaboration with food science and nutriology to develop precise, standardized novel functional foods and Foods for Special Medical Purposes tailored to different clinical scenarios. (4) Conducting Rigorous Clinical Research. Execute well-designed clinical studies to accumulate high-level, evidence-based medical proof and establish clinical guideline recommendations. The ultimate goal is to achieve a full-chain translation from traditional empirical knowledge to modern clinical practice and from experimental discoveries to practical application, thereby providing safe and effective complementary strategies for the integrative management of LC. Through these concerted efforts, it is anticipated that LC prevention and treatment will advance from an experience-based model towards precision and personalized medicine, ultimately improving patients’ quality of life and realizing the clinical value of integrated Chinese and Western medicine in comprehensive cancer care.
